# Supplementary material for: The impact of the reform of rural collective property rights system on villagers’ public participation: An empirical study based on CRRS 2020 data
Source: PLoS One. 2025 Jan 27;20(1):e0316899. doi: 10.1371/journal.pone.0316899 (PMC11771896; doi:10.1371/journal.pone.0316899)
Supplement: Attachment — Submitted filename: Response to Reviewers.docx [file pone.0316899.s001.docx]

**Title:** The impact of the reform of rural collective property rights system on villagers' public participation: An empirical study based on CRRS 2020 data (Manuscript ID: PONE-D-24-33936)

# **Point-by-point response to reviewers**

# Reviewer #1:

**Comment 1:** The first paragraph of this article is not well written, it is too "Chinese". As an international magazine, it is very important to propose the research topic of this article from an international perspective, but this article does not do this.

**Reply:** Thanks for the constructive comments and we are gladly take the suggestion. Based on your suggestions, we have reconsidered and written the Introduction section, with the main modifications as follows:

(1)The first paragraph focuses on public participation, emphasizing its significance and the widespread attention given to it in existing research. However, it also points out that the effectiveness of public participation in rural areas of some countries, such as the United States, Cameroon, Vietnam, and China, which is the focus of this study, is still unsatisfactory. It also highlights the necessity of actively guiding villagers’ public participation .

(2)The second to fourth paragraphs emphasize the importance of the ongoing reform of collective property rights system in China, as well as the focus and limitations of existing research.

(3)Based on the above analysis, the fifth and sixth paragraphs propose the research question of this study: Can the reform of the collective property rights system effectively increase villagers' public participation? Additionally, incorporating other suggestions for revision, we have also included the conclusions and contributions of this study.

These revisions aim to make the opening paragraph clearer, logically more coherent, and relevant to international audiences and the specific context of China. Thank you again for your insightful comments.

The following are the revised parts (marked in red).

# 1. Introduction

Public participation, serving as a pivotal force in driving social progress and democratization, has garnered widespread acknowledgment for its significance[1,2]. Various types of public participation, such as voting, engaging in public decision-making, negotiating and discussing and providing volunteer services[3–5], not only effectively convey the needs of the public and ensure that policymaking is scientific and reasonable, but also stimulate the vitality and creativity of various sectors of society, thereby earning widespread attention and research. For instance, public participation can facilitate the sustainable development of the environment, local urban planning, the formulation of transportation policies, and numerous other fields. [6–10], while public apathy will be a critical barrier to public affairs[11]. However, despite the immense potential and value demonstrated by public participation in practice, its effectiveness faces challenges in some rural areas. For example, the old rural economy of farming in the United States presents conservative public participation[12]. Of course, this has also been confirmed by rural areas in developing countries such as Cameroon and Vietnam[13,14]. In rural China, rapid economic development and substantial migrant for work have led to profound changes in the social structure [15,16]. Families and individuals are gradually detaching from the village collective, tending towards individualization, leading to issues such as loose management of rural public affairs and indifference in interpersonal relationships[17]. These problems have further exacerbated the difficulty of democratic governance in rural society, posing unprecedented challenges. Therefore, how to actively guide villagers to participate in village public affairs has become particularly important. This will not only enhance the efficiency of public participation in rural China, but also have far-reaching implications for promoting comprehensive social progress in other countries facing similar issues.

The ongoing rural collective property rights system reform in China represents the fourth major reform practice and innovation after land reform, collectivization, and household contract responsibility system[18,19]. Since its implementation in 2014[20], this reform has set the goal of “establishing a well-defined, fully empowered, smoothly transferable, and strictly protected rural collective property rights system with Chinese characteristics, and protecting and developing the legitimate rights and interests of villagers as members of rural collective economic organizations” [21] . By establishing a clear collective property rights system and operating collective assets in a market-oriented manner, a large amount of idle rural collective resource elements can be activated[22]. From 2014 to 2021, after five batches of pilot projects for this reform, the national inventory and verification of collective book assets amounted to 7.7 trillion yuan, including 3.5 trillion yuan of operating assets [23].

The overall progress of the reform has been smooth. Many scholars have explored from the perspective of economic benefits and pointed out that, from a micro perspective, the reform has enhanced the vitality of collective economic organizations, promoted collective income generation and increased villagers' income. From a macro perspective, the reform has boosted productivity and further activated new driving forces for rural development in China[19,24].

It is noteworthy that the governance effects of the reform of the rural collective property rights system have also begun to attract attention. This reform focuses on and revolves around the shareholding cooperative system, which entails quantifying and allocating collective operational assets to individuals and households on the basis of asset inventory and membership determination. Consequently, the reform has facilitated the reconstruction of power relations in villages, empowering villagers with more political and economic rights, enhancing their awareness and capacity to engage in rural public affairs, and enriching the effective forms of deliberative democracy in villages[25,26]. A study, utilizing survey data from 1657 villagers in 87 villages across 18 provinces in China in 2018, analyzes the impact of this reform on village democracy and find that the reform strengthens rural democracy and significantly promotes democratic governance in villages [27]. However, this study also has its limitations, as the independent variable of the reform of the collective property rights system is measured by villagers' responses to whether the reform has been completed.

Therefore, based on the actual situation in rural China, this study aims to more accurately measure the reform as well as villagers’ public participation, in order to conduct better empirical analysis. The findings of this study reveal that the reform of the rural collective property rights system has significantly promoted the public participation of villagers, especially in terms of total participation, interest expression, and election voting. However, its impact on volunteer service is limited. Mechanism analysis reveals that reform promotes villagers’ public participation by reducing migration for work. Heterogeneity analysis emphasizes the reform has greater impact on suburban villages and villages with better collective economy. Further analysis indicates villagers have heard that reform can significantly increase their public participation. Suggestions of this study include further deepening the reform of rural collective property rights system, promoting local employment and entrepreneurship, implementing the reform differently based on actual village conditions and increasing the promotion and guidance efforts for the reform.

The contributions of this study are threefold. Firstly, use more accurate indicators to measure the reform of rural collective property rights system and public participation. For the reform of the rural collective property rights system, this study measures whether the reform has been completed based on direct question from village level questionnaires, while previous studies verified the completion of the reform through villagers' responses; for public participation, this study establishes three main indicators and one comprehensive indicator, namely interest expression type, election voting type, volunteer service type and total participation. In comparison, previous studies mostly used only one variable for measurement. Secondly, the multi-level linear regression model and other econometric analysis methods used in this study scientifically and comprehensively investigated the impact of the reform on villagers' public participation, as well as the mechanism and heterogeneity. Thirdly, in terms of data, this study used data from 284 village committee questionnaires and 7451 village questionnaires from 10 provinces in China, which is also authoritative and representative. Above of these contributions help proposing countermeasures and suggestions on how to better promote the public participation of villagers in the process of deepening reform.

**Comment 2:** The literature review and research hypothesis of this article are put together, and this arrangement does not seem to be logical. Generally, the literature review is carried out in the introduction part, or the literature review is delayed. The hypothesis is put together with the theoretical mechanism, and the second paragraph of the current article should be more appropriate as a theoretical mechanism and research hypothesis.

**Reply:** Thank you very much for providing detailed advice and guidance. The title of the second part does not match its content well and needs to be revised. We have changed the title to “Theoretical analysis and research hypothesis” and made modifications in this part.

The following are the revised parts (marked in red).

# **2. Theoretical analysis and research hypothesis**

The reform of the rural collective property rights system encompasses three key aspects: strengthen the management of rural collective assets, carry out property rights reform of operational assets, exploring the forms of realizing rural collective economy (Fig 1)[21]. Throughout the foundational work of this reform, the principle of respecting villagers’ will is paramount. This principle entails harnessing villagers as the primary agents, fostering their innovation and creativity, granting them the right to choose, and ensuring their rights to information, participation, expression, and supervision. Ultimately, this transforms villagers into active participants and beneficiaries of the reform.

**Fig 1. The content of the reform of rural collective property rights system.**

Consequently, the reform of the rural collective property rights system will affect villagers’ public participation in three ways: firstly, by strengthening the management of rural collective assets through various measures, it establishes a foundation for villagers to engage in public participation. Experiences from other developing countries indicate that such approaches not only elevate villagers’ awareness and trust in collective assets but also ignite their enthusiasm for participating in public affairs [28,29]. Secondly, promoting the rural collective management asset shareholding cooperative system guarantees villagers’ shareholding rights as members of collective economic organizations[30]. This enables villagers to participate more directly in the decision-making and operations of the collective economy, thereby expanding the channels and depth of their public participation. Lastly, exploring effective collective economy models, encouraging villager innovation, and supporting the standardized transfers and transactions of rural property rights not only promote the diversified development of rural economy, but also offer more platforms and opportunities for villagers’ public participation [31,32]. By ensuring villagers’ right to know, participate, express and supervise in the reform process, the transformation from villagers being mere observers to active participants and beneficiaries of the reform can be truly realized.

Based on the above analysis, this study presents research hypothesis 1:

Hypothesis 1 (H1): The reform of rural collective property rights system can promote the villagers’ public participation.

The positive reshaping of rural social structures through the rural collective property rights system cannot be overlooked. Research shows that the reform can effectively attract villagers who work outside to return home[33]. Behind this trend lies villagers’ recognition and expectation of the development potential of their hometown, as well as their strong pursuit of personal rights and a sense of belonging. Returning villagers not only bring rich work experience and entrepreneurial enthusiasm, but also inject new vitality into rural communities, alleviating the issue of rural hollowing and providing a solid foundation for broader public participation among villagers[34,35]. In the process of public participation, villagers can not only express their own demands and interests more directly, but also jointly promote the harmony and development of rural society through collective action, achieving a win-win situation for both individuals and the collective.

Therefore, the hypothesis 2 can be proposed:

Hypothesis 2 (H2): The reform of rural collective property rights system reduces the possibility of migration for work, thereby promoting villagers’ public participation.

The impact of collective property rights system reform on villagers’ public participation will be influenced by factors such as the geographical location and economic development level of the village. Firstly, based on the distance from the city center, rural areas can be divided into suburban villages and general villages[36]. Compared with general villages, suburban villages have their own development and governance characteristics. In terms of development, suburban villages have access to more resources and are more likely to take the lead in achieving development goals[37,38]. In terms of governance, suburban villages are often regarded as complex governance units with intertwined interests and mixed personnel, making them more prone to conflicts[39]. But it has also received more attention and policy tilt. The reform of the collective property rights system emphasizes the need for suburban villages to actively establish and improve rural collective economic organizations, and promote the reform of the shareholding cooperative system for operating assets.

Similarly, previous studies have shown that well-developed collective economy not only provide a more solid material foundation and impetus for rural areas, but also greatly enhance villagers’ profound understanding and strong sense of belonging towards the value of collective assets[40,41]. The deepening of this sense of belonging and cognition has laid a solid mass foundation for the smooth progress of rural collective property rights system reform. In this context, with the deepening of the reform of the collective property rights system, it is possible to more accurately and effectively clarify property rights relationships, effectively protect the legitimate rights and interests of villagers, and greatly stimulate their enthusiasm and initiative to public participation.

Thus, it is safe to propose the following hypotheses:

Hypothesis 3(H3): Compared to general villages, the reform of the collective property rights system in suburban villages has a more significant promoting effect on villagers’ public participation.

Hypothesis 4 (H4): The better the rural collective economy, the more obvious the promotion effect of the reform of the collective property rights system on villagers’ public participation.

**Comment 3:** In the empirical part, the current empirical part lacks theoretical depth. It is recommended to combine the hypotheses in the previous article for a more in-depth analysis to increase the theoretical depth.

**Reply:** Thank you very much for your insightful and constructive comment. We have revised the empirical part according your suggestion. In the revised version, we have combined the empirical analysis results with the analysis in “2. Theoretical analysis and research hypothesis” for a more detailed explanation.

The following are the main revised parts (marked in red).

# 4. Empirical results and analysis

## 4.1 Benchmark regression results

As shown in Table 4, it can be observed that after controlling for possible related variables, the reform of rural collective property rights system has a significant positive impact on villagers’ total public participation (β=0.102, p≤0.1), as well as on interest expression (β=0.044, p≤0.1) and election voting (β=0.076, p≤0.05). However, the impact of the reform on villagers’ participate in volunteer service is not significant. Overall, the results of the benchmark regression indicate that the reform of rural collective property rights system is conducive to promoting villagers’ public participation, and hypothesis 1 has been validated.

The aforementioned results are primarily attributed to the economic incentives stemming from clear property rights and the enhancement of rights awareness[48], as mentioned in the analysis of H1. Due to the reform of rural collective property rights system, villagers have become increasingly attentive to the distribution of collective benefits, which has promoted an increase in the overall public participation, manifested as broader willingness and actions to participate. Among them, the notable increase in participation in interest expression and election voting may be due to the reform providing villagers with clearer channels for their interests and a fairer election environment, thereby enhancing their willingness and ability to express their opinions and exercise their rights.

However, the participation in volunteer service has not increased significantly, which may be because volunteer services are mostly based on individual voluntarism and dedication[49]. Although the reform has enhanced villagers’ awareness of economic rights, it may not directly translate into motivation for voluntary service. Furthermore, volunteer service requires a certain degree of organizational mobilization and social atmosphere[50], and during the reform process, insufficient attention may have been given to these non-economic incentives. In summary, future reform should comprehensively consider both economic incentives and sociocultural factors, promoting villagers’ comprehensive public participation from multiple dimensions.

## 4.3 Mechanism analysis

In order to investigate in depth the channels through which the reform of the collective property rights system affects the villagers’ public participation, this study constructs a mediation effect model to test research hypothesis H2. Firstly, examine the effect of the independent variable on the dependent variable, then examine the effect of the independent variable on the mediator variable, and finally examine the combined effect of the independent and mediator variables on the dependent variable. The analysis results are shown in Table 7.

Column (1) is the same as the first column in Table 4, indicating that the reform of the collective property rights system improves the villagers’ public participation (β=0 102, p ≤ 0.1). Column (2) shows that the reform significantly reduces the probability of villagers migration for work(β=-0) 062, p ≤ 0.1). Column (3) suggests that including migration for work in the regression equation of the Column (1) still enhances the level of villages’ public participation (β=0 104, p ≤ 0.1). This indicates that migration for work plays a mediating role in the influence of the reform of the collective property rights system on villagers’ public participation, and hypothesis, verifying Hypothesis H2.

Reform often accompanies the development and growth of collective economy, providing villagers with more employment opportunities and sources of income[51]. This has to some extent reduced the willingness and probability of migration for work, enabling more villagers to stay locally and have more time and energy to invest in village governance. Retaining villagers not only enhances the village's human capital but also provides more opportunities for organizing and implementing public participation activities. In summary, the reform of the rural collective property rights system effectively elevates villagers’ public participation by reducing the probability of them migration for work.

## 4.4 Heterogeneity analysis

The previous analysis verified the impact of the reform of rural collective property rights system on villagers’ public participation. However, the social situation of different villages will have varying impacts on the effectiveness of the reform. Therefore, this study uses whether rural areas are located in the suburban areas as a proxy variable for rural geographical location, and uses the rural collective economy as a proxy variable for the rural economy to further conduct heterogeneity analysis.

Table 8 presents the results of heterogeneity analysis. From the perspective of urban proximity(first two columns), compared to general villages(β=0.075, p>0.1), the reform has a greater impact on the villagers’ public participation in suburban villages(β=0.135, p≤0.05). Suburban villages are more susceptible to urban radiation. Study has shown that urban residents have a higher willingness in public participation[52]. Therefore, for rural areas located around cities, the enthusiasm of villagers’ public participation after the reform of the collective property rights system is further stimulated.

From the perspective of the rural collective economy (last two columns), compared to villages with a collective economic development level of less than or equal to that of the average(β=0.074, p>0.1), the reform of the collective property rights system has a greater impact on the villagers’ public participation with a collective economic development level greater than that of the average(β=0.231, p≤0.05). Therefore, the effect of stimulating villagers’ public participation through reform is more significant in villages with better economic development levels[53]. Therefore, hypotheses 3 and 4 have also been verified to be correct.

## 4.5 Further analysis

Finally, a significant endeavor of this study lies in the fact that, in previous research, whether the reform of the collective property rights system was completed was mostly confirmed through villagers’ responses. Therefore, in this section, the independent variable in the benchmark regression, which was originally based on official answers indicating whether the village had completed the reform, is replaced with individual villagers’ responses, to further analyze the scientific validity of this substitution. Table 8 presents further analysis results.

Column (1) presents the regression results for the entire sample based on the question, “Have you heard of the reform of the collective property rights system”, which serves as a proxy variable for the reform. The regression results indicate that having heard of the reform significantly enhances villagers’ total public participation(β=0.087, p≤0.01).

Column (2) shows the regression results for the subset of samples where the village-level reform of the collective property rights system has been completed (i.e., Reform completed=1), using the same question as in Column (1). The results again demonstrate that having heard of the reform significantly boosts villagers’ total public participation (β=0.081, p ≤ 0.01).

Column (3), however, presents the regression results based on the question, "Do you know the rural collective property rights system reform is currently underway (or has already been completed) in this village?". Surprisingly, the results indicate that villagers who have heard of the village-specific reform do not have a significant impact on their total public participation, contrary to the previous conclusions[27].

These discoveries are very interesting and of great significance. On the one hand, it emphasizes the importance of information transparency and policy promotion[54]. When villagers fully understand the reform policies, they are better able to clarify their own rights and responsibilities, actively express their interests and demands, and participate in social affairs[55]. On the other hand, the previous use of villagers’ awareness of reforms as a basis for studying the effectiveness of collective property rights system reform needs to be approached with caution and deserves further discussion.

**Comment 4:** Although the policy implications are mentioned in the last part of this article, they are too weak and the content is too little. It is recommended to propose more targeted policies based on the quantitative results to highlight the practical significance of this article's research.

**Reply:** Thank you for your detailed suggestions. The previous policy implications not only have the weak and insufficient content you pointed out, but they are also a whole paragraph in form, lacking clarity. We have reorganized this section and proposed corresponding policy implications with practical guidance based on research conclusions. In addition, the segmented format makes the presentation clearer and more organized.

The following are the main revised parts (marked in red).

## 5.2 Policy implications

Based on the above conclusions, this study proposes the following policy recommendations. Firstly, further deepen the reform of rural collective property rights system. The core finding of this study is that the reform of rural collective property rights system serves as a crucial prerequisite and powerful impetus for promoting villagers’ public participation. After years of reform efforts, China has established a relatively mature reform approach. What makes this conclusion even more practically significant is the promulgation of the “Law of the People's Republic of China on Rural Collective Economic Organizations” in June 2024, which responds to and solves many questions arising during the reform process from a institutional perspective and will officially take effect in January 2025.[56]. However, according to existing surveys, the coverage of the reform in China still needs further improvement[57]. Therefore, leveraging the existing legal support, it is imperative to persist in and deepen this reform direction, continually enhancing the property rights system and clearly delineating villagers’ rights and interests in collective assets, thereby further stimulating their enthusiasm and motivation in public participation.

Secondly, promote local employment and entrepreneurship. The mechanism by which the reform of rural collective property rights system enhances villagers’ public participation provides an important direction for policymakers: to create more employment and entrepreneurial opportunities for villagers. While advancing the reform, it is necessary to intensify support for rural collective economy. This involves not only providing more financial support but also introducing preferential policies to incentivize and assist rural collective economic organizations in strengthening their economic capabilities. Specific measures include developing industries with distinctive local characteristics, optimizing existing industrial structures, and introducing advanced science and technology[58]. Of course, the government also needs to increase investment in rural infrastructure construction and public service systems, continuously improving rural production and living environments, in order to attract migrant workers to return home for employment or self-employment. As a result, villagers’ willingness to migration for work will decrease, enabling them to enjoy the benefits of the rural collective property rights reform while having more time and energy to participate in public affairs, thereby promoting the overall level of public participation.

Thirdly, implement the reform differently based on actual village conditions. In the process of formulating and implementing reform, full consideration must be given to the differences in geographical location and economic development levels. For villages located in the suburbs of cities and those with higher levels of economic development, reforms should be further deepened and expanded, fully leveraging their demonstration and leading roles in enhancing villagers’ public participation. Meanwhile, for villages in relatively remote geographical locations and those with lower levels of economic development, they should be encouraged to closely integrate with their own realities and actively explore reform paths and governance models that suit their unique characteristics, aiming to achieve precision and effectiveness in reform and development, and promote comprehensive rural revitalization.

Fourthly, increase the promotion and guidance efforts for the reform. Government should intensify its efforts in promoting policies concerning the reform of the rural collective property rights system and emphasize the transparency of information dissemination. Through a variety of channels and methods, including convening villager assemblies for detailed explanations, distributing promotional brochures, and publishing interpretations on online platforms, the government should comprehensively disseminate information about the reform policies, objectives, and their significance to villagers. The objective is not only to raise villagers’ awareness of the reform but also to ensure they have a deep understanding of the tangible benefits it can bring. Additionally, the government needs to strengthen policy guidance, encouraging villagers to actively participate in social governance activities, particularly volunteer service activities, in order to promote comprehensiveness and diversity in public participation. Furthermore, it is necessary to explore effective incentive and safeguard mechanisms to ensure that villagers can genuinely engage in and benefit from the reform and governance process.

**Comment 5:** In terms of language, the language of this article currently has grammatical errors and “Chinese English” phenomena. It is recommended to reorganize the language.

**Reply:** Thank you for your constructive comments on the language of our article. We understand that grammatical errors and “Chinese English” phenomena can detract from the clarity and professionalism of writing.

We have carefully reviewed and revised the language of the article, corrected any grammatical errors and made efforts to eliminate “Chinese English” expressions, such as changing from “migrant work” to “migration for work”, ensuring that the manuscript is clear, concise, and professional.

We appreciate your attention to detail and are grateful for your suggestions, which have helped us improve the overall quality of our writing. We hope that the revised version, which marked in red, meets your expectations and standards.

Reviewer #2:

**Comment 1:** The summary of the paper is clear and reasonable, although the authors might consider refining the last section, e.g., it might be better to describe the contributions of the paper rather than to list policy implications.

**Reply:** Thank you very much for your valuable and detailed comment. Based on your suggestion, we have added the contributions of the paper to the summary part.

The following are the revised parts (marked in red).

# **Abstract**

The reform of rural collective property rights system is of great significance for protecting the collective asset rights and interests of villagers, activating rural resource elements, and achieving rural revitalization. This study is based on 284 village committee questionnaires and 7451 villager questionnaires from 10 provinces in China, and uses multi-layer linear regression models to explore the impact of the reform of rural collective property rights system on villagers' public participation. Research has found that:(1) the reform of rural collective property rights system that has been completed at the rural level can significantly enhance the public participation of villagers, including total participation (β=0.102, p ≤ 0.1), interest expression (β=0.044, p ≤ 0.1), and election voting (β=0.076, p ≤ 0.05). However, the impact on volunteer service is not significant (β=-0.004, p>0.1); (2)mechanism analysis shows that the reform can reduce the probability of migration for work, and thus enhance the level of public participation; (3)heterogeneity analysis reveals that the reform has a greater impact on the villagers’ public participation in suburban villages and villages with better collective economy; (4)further analysis discovers that villagers have heard that reform can significantly enhance their public participation. This study comprehensively explores the spillover effects of the reform at the village level on public participation at the individual level through the use of more accurate measurement indicators, empirical analysis methods, and large-scale authoritative data, providing strong support for proposing strategies to promote villages’ public participation.

**Comment 2:** The format used by the author is not conducive to the reading of the article, and it is recommended that the author use a two-sided aligned layout, which may be more helpful.

**Reply:** Thank you very much for providing detailed advice and guidance. Based on this suggestion, we have recognized that the left aligned layout affects the reading experience and have made modifications to two-sided aligned layout of the entire manuscript.

**Comment 3:** The content of the introduction section is reasonable as a whole, but the lack of an author's summary of the paper's findings and a statement of the value of the paper's research in the introductory section may be incomplete.

**Reply:** Thank you for acknowledging the arrangement of the introduction and pointing out the shortcomings. Based on your suggestions, we have added the findings and three contributions of the study, which is also have pointed in the **Comment 7**, “the current ‘6. Conclusion’ might be more appropriate in the introduction”.

The following are the revised parts (marked in red).

Therefore, based on the actual situation in rural China, this study aims to more accurately measure the reform as well as villagers’ public participation, in order to conduct better empirical analysis. The findings of this study reveal that the reform of the rural collective property rights system has significantly promoted the public participation of villagers, especially in terms of total participation, interest expression, and election voting. However, its impact on volunteer service is limited. Mechanism analysis reveals that reform promotes villagers’ public participation by reducing migration for work. Heterogeneity analysis emphasizes the reform has greater impact on suburban villages and villages with better collective economy. Further analysis indicates villagers have heard that reform can significantly increase their public participation. Suggestions of this study include further deepening the reform of rural collective property rights system, promoting local employment and entrepreneurship, implementing the reform differently based on actual village conditions and increasing the promotion and guidance efforts for the reform.

The contributions of this study are threefold. Firstly, use more accurate indicators to measure the reform of rural collective property rights system and public participation. For the reform of the rural collective property rights system, this study measures whether the reform has been completed based on direct question from village level questionnaires, while previous studies verified the completion of the reform through villagers' responses; for public participation, this study establishes three main indicators and one comprehensive indicator, namely interest expression type, election voting type, volunteer service type and total participation. In comparison, previous studies mostly used only one variable for measurement. Secondly, the multi-level linear regression model and other econometric analysis methods used in this study scientifically and comprehensively investigated the impact of the reform on villagers' public participation, as well as the mechanism and heterogeneity. Thirdly, in terms of data, this study used data from 284 village committee questionnaires and 7451 village questionnaires from 10 provinces in China, which is also authoritative and representative. Above of these contributions help proposing countermeasures and suggestions on how to better promote the public participation of villagers in the process of deepening reform.

**Comment 4:** The authors present the hypothesis of this paper in the second part, therefore the title is inappropriate, perhaps “Literature Review and Hypothesis”would be more appropriate.

**Reply:** Thank you very much for your constructive comment. The title of the second part does not match its content well and needs to be revised.

We chose “Theoretical analysis and research hypothesis” as the title for this section because it reflects our approach to building a theoretical framework based on the literature review and then deriving the research hypothesis.

We acknowledge that there may be multiple valid ways to title this section, and we appreciate your suggestion. To address your concern, we would be happy to discuss this further and consider any additional suggestions you may have.

Additionally, we have also revised the titles of the entire manuscript based on **Comment 7**. Please refer to the reply to **Comment 7** for details.

**Comment 5:** The three-line table format is not standardized, and it is recommended that authors use a standardized three-line table for the presentation of tables. And each table is different, it is recommended that the standardized three-line table be used consistently.

**Reply:** Thank you for your valuable and detailed comments. We have revised the 9 tables in the entire manuscript into standardized three-line tables based on your comments.

The following are the main revised Table 1 and Table 4.

**Table 1. Basic frequency and percentage of public participation.**

| **Type** | **Variables** | **Definition** | **Mean** | **S.D.** | **Min** | **Max** |
| --- | --- | --- | --- | --- | --- | --- |
| Total public participation | Aggregate public participation types | Number of public participation types | 2.307 | 0.652 | 0 | 3 |
| Interest expression type | Participate in village assembly | Participation=1, otherwise=0 | 0.867 | 0.339 | 0 | 1 |
| Election voting type | Participate in election voting | Someone from your family is voting in the village committee election=1, otherwise=0 | 0.463 | 0.499 | 0 | 1 |
| Volunteer service type | Participate in crisis rescue efforts | When a crisis occurs in the village, you participate in the rescue as soon as possible=1, otherwise=0 | 0.709 | 0.454 | 0 | 1 |

**Table 4. Benchmark regression results.**

| **Variable** | **(1)** | **(2)** | **(3)** | **(4)** |
| --- | --- | --- | --- | --- |
|  | **Total public participation** | **Interest expression type** | **Election voting type** | **Volunteer service type** |
| Reform | 0.102* | 0.044* | 0.076** | -0.004 |
|  | (0.054) | (0.026) | (0.037) | (0.030) |
| Terrain | 0.089* | -0.024 | 0.075** | 0.014 |
|  | (0.053) | (0.026) | (0.037) | (0.030) |
| Government distance | -0.000 | -0.001 | 0.001 | -0.001 |
|  | (0.001) | (0.001) | (0.001) | (0.001) |
| One Shoulder Multitasking | -0.028 | 0.058* | -0.122*** | -0.010 |
|  | (0.061) | (0.029) | (0.035) | (0.034) |
| Village secretary education | 0.028 | 0.002 | 0.028 | -0.006 |
|  | (0.027) | (0.013) | (0.019) | (0.016) |
| Age | 0.001* | 0.000 | 0.000 | -0.000* |
|  | (0.000) | (0.000) | (0.000) | (0.000) |
| Gender | -0.003 | -0.006 | 0.002 | -0.006 |
|  | (0.013) | (0.007) | (0.010) | (0.010) |
| Education | 0.012* | 0.009** | 0.006 | 0.004 |
|  | (0.007) | (0.004) | (0.005) | (0.005) |
| Party | -0.001 | 0.051*** | -0.049*** | 0.061*** |
|  | (0.021) | (0.012) | (0.016) | (0.016) |
| Income | -0.004 | 0.005 | -0.015*** | 0.037*** |
|  | (0.007) | (0.004) | (0.005) | (0.005) |
| Province | Yes | Yes | Yes | Yes |
| Constant | 2.076*** | 0.733*** | 0.486*** | 0.562*** |
|  | (0.171) | (0.085) | (0.111) | (0.105) |
| N(L1) | 7451 | 7451 | 7451 | 7451 |
| N(L2) | 284 | 284 | 284 | 284 |

Standard errors in parentheses; * p < 0.1, ** p < 0.05, *** p < 0.01

**Comment 6:** The section on policy recommendations is too long, and it is recommended that the authors appropriately segment the content of the article, which would be more helpful for readers to understand the study.

**Reply:** Thank you for your suggestions to optimize this section. Based on your suggestions and the conclusions of this study, we have revised and segmented the policy recommendations section.

The following are the main revised parts (marked in red).

## 5.2 Policy implications

Based on the above conclusions, this study proposes the following policy recommendations. Firstly, further deepen the reform of rural collective property rights system. The core finding of this study is that the reform of rural collective property rights system serves as a crucial prerequisite and powerful impetus for promoting villagers’ public participation. After years of reform efforts, China has established a relatively mature reform approach. What makes this conclusion even more practically significant is the promulgation of the “Law of the People's Republic of China on Rural Collective Economic Organizations” in June 2024, which responds to and solves many questions arising during the reform process from a institutional perspective and will officially take effect in January 2025.[56]. However, according to existing surveys, the coverage of the reform in China still needs further improvement[57]. Therefore, leveraging the existing legal support, it is imperative to persist in and deepen this reform direction, continually enhancing the property rights system and clearly delineating villagers’ rights and interests in collective assets, thereby further stimulating their enthusiasm and motivation in public participation.

Secondly, promote local employment and entrepreneurship. The mechanism by which the reform of rural collective property rights system enhances villagers’ public participation provides an important direction for policymakers: to create more employment and entrepreneurial opportunities for villagers. While advancing the reform, it is necessary to intensify support for rural collective economy. This involves not only providing more financial support but also introducing preferential policies to incentivize and assist rural collective economic organizations in strengthening their economic capabilities. Specific measures include developing industries with distinctive local characteristics, optimizing existing industrial structures, and introducing advanced science and technology[58]. Of course, the government also needs to increase investment in rural infrastructure construction and public service systems, continuously improving rural production and living environments, in order to attract migrant workers to return home for employment or self-employment. As a result, villagers’ willingness to migration for work will decrease, enabling them to enjoy the benefits of the rural collective property rights reform while having more time and energy to participate in public affairs, thereby promoting the overall level of public participation.

Thirdly, implement the reform differently based on actual village conditions. In the process of formulating and implementing reform, full consideration must be given to the differences in geographical location and economic development levels. For villages located in the suburbs of cities and those with higher levels of economic development, reforms should be further deepened and expanded, fully leveraging their demonstration and leading roles in enhancing villagers’ public participation. Meanwhile, for villages in relatively remote geographical locations and those with lower levels of economic development, they should be encouraged to closely integrate with their own realities and actively explore reform paths and governance models that suit their unique characteristics, aiming to achieve precision and effectiveness in reform and development, and promote comprehensive rural revitalization.

Fourthly, increase the promotion and guidance efforts for the reform. Government should intensify its efforts in promoting policies concerning the reform of the rural collective property rights system and emphasize the transparency of information dissemination. Through a variety of channels and methods, including convening villager assemblies for detailed explanations, distributing promotional brochures, and publishing interpretations on online platforms, the government should comprehensively disseminate information about the reform policies, objectives, and their significance to villagers. The objective is not only to raise villagers’ awareness of the reform but also to ensure they have a deep understanding of the tangible benefits it can bring. Additionally, the government needs to strengthen policy guidance, encouraging villagers to actively participate in social governance activities, particularly volunteer service activities, in order to promote comprehensiveness and diversity in public participation. Furthermore, it is necessary to explore effective incentive and safeguard mechanisms to ensure that villagers can genuinely engage in and benefit from the reform and governance process.

**Comment 7:** The “5. Discussion” section is overloaded with conclusions, policy implications and discussion, of which the conclusions and policy implications should be placed in “6. Conclusions and policy implications”, whereas the current “6. Conclusion” might be more appropriate in the introduction. It is recommended that the authors re-frame the content of the article and use the correct title to match the content.

**Reply:** Thank you very much for your constructive and detailed suggestions. Based on your suggestions, we have realized that there are significant shortcomings in the arrangement of the manuscript. Therefore, we have reconsidered and rearranged the title of the entire manuscript. Of course, as mentioned in the reply to **Comment 3**, the original “6. Conclusion” has been placed in the introduction.

The following are the revised level 1 heading and level 2 heading.

# **1. Introduction**

# **2. Theoretical analysis and research hypothesis**

# **3. Research design**

## 3.1 Data sources

## **3.2 Variable selection**

# **4. Empirical results and analysis**

## **4.1 Benchmark regression results**

## **4.2 Robustness test**

## **4.3 Mechanism analysis**

## **4.4 Heterogeneity analysis**

## **4.5 Further analysis**

# **5. Conclusions and policy implications**

## **5.1 Conclusions**

## **5.2 Policy implications**

## **5.3 Limitations and future research directions**

Finally, thanks again for the efforts to make this paper the best it can be. Many thanks for the affirmation and approval of the article.

With best regards

Yours the Author

November 28, 2024
